# Effect of different surface treatments on the repair bond strength between aged and new alkasite based restorative material

**DOI:** 10.12688/f1000research.148326.2

**Published:** 2024-06-06

**Authors:** Tina Puthen Purayil, Kishore Ginjupalli, Kalyana Pentapati, Aastha Dureja, Rashmi Nayak

**Affiliations:** 1 Conservative Dentistry and Endodontics, Manipal College of Dental Sciences, Manipal, Manipal Academy of Higher Education, Manipal, Karnataka, 576104, India; 2 Dental Materials, Manipal College of Dental Sciences, Manipal, Manipal Academy of Higher Education, Manipal, Karnataka, 576104, India; 3 Public Health Dentistry, Manipal College of Dental Sciences, Manipal, Manipal Academy of Higher Education, Manipal, Karnataka, 576104, India; 4 Conservative Dentistry and Endodontics, D.Y. Patil University School of Dentistry, Nerul, Navi Mumbai, Maharashtra, 400706, India; 5 Pedodontics and Preventive Dentistry, Manipal College of Dental Sciences, Manipal, Manipal Academy of Higher Education, Manipal, Karnataka, 576104, India

**Keywords:** Alkasite, Bond strength, Repair, Surface treatment

## Abstract

**Background:**

This study investigates various surface treatment methods to assess shear bond strength between set Cention N (alkasite-based restorative material) and new alkasite based restorative material. Assessing different surface treatments provide insights in optimizing repair procedure that enables durability of the restoration, thus potentially benefitting clinical outcomes.

**Methods:**

A total of 48 alkasite based restorative material blocks, measuring 4 mm in depth and 4 mm in diameter, were prepared. The samples were randomly divided into 8 groups (n = 6) according to the surface treatment done. Group I: Surface preparation by bur; Group II: Surface treatment by laser; Group III: Application of 2-step etch and rinse adhesive (Adper Single Bond 2 adhesive),Group IV: Application of single step self-etch adhesive (Scotchbond Universal adhesive); Group V: Bur preparation followed by application of 2-step etch and rinse adhesive; Group VI: Bur preparation followed by application of single step self-etch adhesive; Group VII: Laser preparation followed by application of 2-step etch and rinse adhesive; and Group VIII: Laser preparation followed by application of single step self-etch adhesive. Post-surface preparation, all the specimens were restored with newly mixed alkasite material. Repair bond strength measurements were assessed with universal testing machine. Shapiro-Wilk and Levene's tests were used to check normality and Homogeneity of variance. ANOVA with post-hoc Games-Howell test and two-way ANOVA with post-hoc Bonferroni test was performed to evaluate the influence of surface preparation on the repair bond strength.

**Results:**

Using a 2-step etch and rinse adhesive resulted in a higher repair bond strength (26.05±2.12) compared to other surface treatments. In contrast, roughening of the surface with burs led to lowest repair bond strength (17.06±3.29) (P=0.02).

**Conclusion:**

Application of 2-step etch and rinse adhesive to the existing alkasite based restorative material provides superior bonding with the newly added alkasite based restorative material.

## Introduction

In the past, complete replacement of restorations was the standard approach for eliminating small defects in restorations. However, complete removal of the restoration followed by preparation of the tooth to receive a new restoration weakens the tooth structure, resulting in unnecessary removal of tooth structure and, in some cases, irreversible injury to the dental pulp. Even in the absence of secondary caries or discoloration at the tooth-restoration interface, repair is suggested instead of complete replacement of the restoration, primarily because of its conservative approach. Thus, repair of the restoration is an effective approach to enhance the longevity of the restorations. ^1^^–^^4^ In addition, durability of the repaired restorations is equivalent to that of replacement of the restorations. ^5^ However, the success or failure of such a process is dependent on the bond strength between the old and the new restorative materials at their interface, which is affected by a myriad of parameters, including surface treatment of the old restoration before placement of new restorative material.

Various methods that alter the physical and chemical features of the surfaces of old restorative materials have been employed to improve the bond strength between the existing and new restorative materials. These include surface preparation using a bur, laser, air abrasion with aluminum oxide particles, pre-treatment of the surface with silane, and the use of resin-based adhesive systems along with different primers. ^6^^–^^8^

Alkasite is a tooth-colored restorative material (Cention ^®^ N, Ivoclar Vivadent Liechtenstein), widely used for deciduous and permanent restorations of Class I, II, and V carious lesions. ^9^ Manufacturers and recent research indicate that it exhibits flexural strength comparable to that of silver amalgam, along with superior handling characteristics. ^9^^,^^10^

As this material is recently introduced, data on the clinical performance and survival or clinical service life of the material along with the type of failure of the material in a variety of clinical conditions are not widely available. However, in the event of failure of such restorations, in the form of dislodgement or fracture of the material, it is important to establish a suitable repair method.

Most surface treatment methods aim to increase surface area of the bonding between the two materials to promote the interaction between the aged and new restorative materials thus improving the bond strength. However, literature on the effect of surface treatment on the bond strength between the old and new alkasite based restorative materials is not available. Hence, the main aim of this study was to evaluate the effect of various surface treatments on the repair bond strength between old and new alkasite based restorative materials.

## Methods

A total of 48 acrylic blocks (4 cm height × 2 cm width) were prepared using a self-cure acrylic resin (DPI-RR Cold Cure, DPI, Mumbai, India). One-day after the polymerization of the acrylic blocks, a cylindrical hole of 4 mm × 4 mm was drilled on one side of the acrylic block.

Alkasite based material powder was weighed using an electronic balance and added to a pre-weighed quantity of liquid at a ratio of 4.6:1, as recommended by the manufacturer. Both the powder and liquid were mixed with a plastic spatula for 30 s to obtain a thick putty-like consistency suitable for packing. The material was packed into the holes in the acrylic block, and curing was performed for 20 s using a Light Emitting Diode (LED) light curing unit at 850 mW/cm ^2^ (3M ESPE Elipar, St Paul, USA). The specimens were then stored in distilled water at 37°C for one month for ageing. ^11^

### Surface preparation

After the aging process, the acrylic blocks containing alkasite based material were randomly divided into eight groups (n=6) and subjected to either of the following surface treatments: Table 1 describes the various methods used for surface preparation across the different groups.

**Table 1.** Description of the various surface preparation methods used in different groups.

Group	Group code	Type of surface preparation	Type of adhesive used
I	Bur	Bur	-
II	Laser	Laser Irradiation	-
III	ASB2	-	2-step etch and rinse adhesive
IV	SBUA	-	Single step self-etch adhesive
V	Bur+ASB2	Bur	2-step etch and rinse adhesive
VI	Bur+SBUA	Bur	Single step self-etch adhesive
VII	Laser+ASB2	Laser Irradiation	2-step etch and rinse adhesive
VIII	Laser+SBUA	Laser Irradiation	Single step self-etch adhesive

**Group I (Bur):** The surface of alkasite based material was roughened using a TR 11 diamond bur (Mani, Tochigi, Japan) with a high-speed handpiece (NSK M25, Nakanishi Inc, Kanuma City, Japan) under water spray. Bur was replaced after surface preparation of five specimens.

**Group II (Laser):** The alkasite based material surface was irradiated by an Er;Cr:YSGG laser (Waterlase, Biolase Technology, Sanclemente, CA, USA) at 2780 nm wavelength at 3 W output power and 20 Hz frequency of 60μsec duration. A laser tip was used with a gentle sweeping motion at a working length of 1 mm with a spot size of 800μm (MZ-8 Ziptip, Biolase) under a water spray.

**Group III (ASB2):** The surface of alkasite based material was treated with 2 step etch and rinse adhesive (Adper Single Bond 2, 3M ESPE St Paul MN, USA) ( Table 2) where the specimen was first etched with 37% phosphoric acid (Ivoclar Vivadent, USA) for 15 s, followed by rinsing for 10 s with water and air drying. Subsequently, two coats of adhesive were applied to the etched surfaces of alkasite based material with gentle agitation, followed by air blowing of the excess adhesive to leave a thin film. This was followed by light-curing of the adhesive for 10 s using an LED light-curing unit.

**Table 2.** Description of the various materials and composition used in the study.

Materials	Composition	Manufacturer
Cention N	Powder: Calcium-fluoro-silicateglass, Barium-Alumino-silicate glass, Ytterbium trifluoride, Isofiller (Copolymer), A copper salt & thiocarbamide-self cure Initiator or Ivocerin and acyl phosphine oxide-photoinitiator, Pigment Liquid: Urethanedimethacrylate, Tetramethyl-xylylendiurethane dimethacrylate, Tricyclodecan-dimethanol dimethacrylate, Polyethylene glycol 400 dimethacrylate, Initiator (Hydroperoxide), Stabilizer	Ivoclar Vivadent Liechtenstein
Adper Single Bond 2	Bisphenol-A glycidyl methacrylate,2-hy-droxyethyl methacrylate, dimethacrylates, polyalkenoic acid copolymer, 10 vol% of 5-nm silica nanofiller, initiators, water, ethanol	3M ESPE St Paul MN, USA
Scotchbond Universal Adhesive	Methacryloyloxydecyl dihydrogen phosphate monomer, dimethacrylate resins, Hydroxyethyl methacrylate, methacrylate functionalized polyalkenoic acid, filler, ethanol	3M ESPE, St Paul, MN, USA

**Group IV (SBUA):** The surface of alkasite based material was treated with a single step self etch adhesive (Scotchbond Universal Adhesive, 3M ESPE, St Paul, MN, USA) ( Table 2) using a microbrush for 20s, thinned by mild air pressure for 5 s, followed by light-curing of the adhesive for 10 s using an LED light-curing unit.

**Group V (Bur+ASB2):** The alkasite based material surface was roughened as described for Group I, followed by application of 2-step etch and rinse adhesive as described for Group III.

**Group VI (Bur+SBUA):** The alkasite based material surface was roughened as described for Group I, followed by application of a single step self-etch adhesive as described for Group IV.

**Group VII (Laser+ASB2):** The alkasite based material surface was laser-irradiated as described for Group II, followed by application of 2-step etch and rinse adhesive as described for Group III.

**Group VIII (Laser+SBUA):** The alkasite based material surface was laser-irradiated as described for Group II, followed by application of a single step self-etch adhesive as described for Group IV.

### Specimen preparation

After the completion of the surface preparation of alkasite based material as described above, a newly mixed alkasite based material as per the manufacturer’s instructions was build up to a height of 4 mm. Subsequently, the material was cured using LED light curing for 20 s. After the curing, samples were stored in distilled water for 24 h.

### Measurement of repair bond strength

The repair bond strength was evaluated in shear mode. The samples were fastened to the lower half of a universal testing machine (Model 3366; Instron Corp., USA). A compressive load was applied at a loading rate of 0.5 mm/min at the interface between the old and new alkasite based materials until debonding. The maximum load observed during the test, divided by the area of the specimen, was reported as the repair bond strength in MPa (n=6).

### Statistical analysis

Data were analyzed using SPSS version 20 (IBM Corp. Released 2011. IBM SPSS Statistics for Windows, Version 20.0. Armonk, NY: IBM Corp.). Statistical significance was set at P<0.05. Normality was tested using the Shapiro-Wilk test. Based on the normality, and test of homogeneity of the variances (Levene statistic) mean were compared using ANOVA with post-hoc Games-Howell test. Subsequently, a two-way ANOVA with post-hoc Bonferroni test for inter-group comparisons was performed to evaluate the role of surface preparation and adhesive application on the repair bond strength. The data for this study are available in Figshare. ^12^

## Results

There was a significant difference in the mean repair bond strength between the eight groups (P=0.02). Post-hoc tests showed that Group III (ASB2) (26.05±2.12) and Group VII (Laser+ASB2) (25.84±2.35) had significantly higher mean repair bond strengths than Group I (Bur). No other significant differences were observed between groups ( Table 3). Two-way ANOVA was used to evaluate the role of surface preparation and adhesive application on repair bond strength. Levene’s test showed no significant differences in variance across groups (P=0.25). There was no significant interaction between the type of adhesive and surface preparation (P=0.121). The main effects showed that the type of adhesives showed a significant difference on the repair bond strength (P=0.01), whereas no significant difference was observed with respect to the surface preparation. The estimated marginal means for Group III (ASB2) were significantly higher than compared to those without adhesives (P=0.003), whereas no other significant differences were observed with respect to adhesives on the repair bond strength.

**Table 3.** Comparison of repair bond strength values among the study groups.

Group	Mean and Standard Deviation (MPa)	P-value	Post-hoc test
I. Bur	17.06±3.29	0.02; Sig	III, VII>I
II. Laser	21.52±4.63		
III. ASB2	26.05±2.12		
IV. SBUA	23.83±5.09		
V. Bur+ASB2	24.29±6.84		
VI. Bur +SBUA	24.84±4.61		
VII. Laser+ASB2	25.84±2.35		
VIII. Laser+SBUA	20.13±5.91		

## Discussion

Failure of dental restorations or the need for retreatment is not uncommon in routine dental practice. In such cases, re-restoration is a standard procedure aimed at repairing and restoring the tooth to its natural form and function. The surface of the existing restoration is prepared using a variety of methods to enhance its bonding with newly placed restoration material, which can influence the bond strength between the two materials, impacting the longevity of the repair. ^13^^,^^14^

Alkasite restorative material is a new category of filling materials based on urethane dimethacrylate (UDMA) resin. It is supplied as a self-curing powder/liquid formulation, along with optional light curing. Its ability to effectively polymerize to complete depth of the restoration leads to a high polymer network density in the material. This could be attributed to the sole use of the cross-linking methacrylate monomer, along with a stable, efficient self-cure initiator. ^9^ The existing literature is scarce in terms of the methods to be followed to achieve superior bonding between the existing restorative material and new material in case of repair. Thus, the present study aimed to investigate the effect of different surface treatments on the repair bond strength between existing and new alkasite based restorative materials.

The results of the present study indicate that the use of adhesive results in a superior repair bond strength. This was in accordance with a study conducted on composites ^15^ where the use of adhesive proved to be a conservative and effective treatment approach for the repair of restorative materials. Among the two adhesive systems, the highest repair bond strength was observed with the application of 2-step etch and rinse adhesive. Among the various surface treatments evaluated in the present study, surface preparation using burs resulted in the lowest repair bond strength.

2-step etch and rinse adhesive is a fifth generation bonding agent that employs a separate etching step prior to the application of adhesive. A separate etching process followed by rinsing would have helped in cleaning the surface of the restoration and increased its surface roughness, leading to an increase in the surface area of the bonding, better wettability of the adhesives, and leading to superior repair bond strength. A similar improvement in bond strength has been reported in previous investigations. ^16^^,^^17^

Single step self-etch adhesive, a seventh generation dentin bonding agent, on the other hand, is a hydrophilic multipurpose and all-in-one adhesive that does not require an additional etching step. Despite its ease of use, it showed less favorable repair bond strength than 2-step etch and rinse adhesive. The absence of a separate etching step, which is generally followed by washing would not result in the exposure of a clean surface with an enhanced surface area needed for superior bonding. In addition, the hydrophilicity of the adhesive may interfere with the durability of the interfacial bond repair because hydrophilic adhesives tend to absorb more water over time, resulting in hydrolytic degradation. Secondly, when universal adhesives with etchant action are applied to enamel or dentin, the buffering capacity of the dental hard tissue neutralizes its acidity. ^18^ In contrast, during the repair of alkasite based material, the acidic resin monomer was not neutralized because of the absence of apatite, which may have resulted in inadequate polymerization of the newly prepared restorative material. ^19^

Er;Cr:YSGG laser was employed in this study because of its effectiveness in ablating restorative surfaces using highly energized water molecules. ^20^ Despite its effectiveness over other lasers in restorative repair, the present study did not show superior repair bond strength. This could be due to the inability of the highly viscous alkasite based material to flow into irregularities created by the laser. Despite both etchant and laser rendering the surface of the restoration rough by varying degrees, the use of etchant followed by application of adhesive resulted in better repair bond strength. This could be due to the better flowability of the adhesive, which provided higher micromechanical retention of the restorative material. ^21^

The use of burs resulted in the least favorable results in terms of the repair bond strength. To ensure a close adaptation of the aged and newly mixed alkasite based material, there is a need for an intermediate material, as the newly mixed material may not properly wet the aged alkasite based material, making it difficult to achieve the bond. The adhesives help in penetrating into the irregularities and optimizing the bond that would otherwise be difficult due to the high viscosity and low wetting potential of the restorative material. ^22^^,^^23^ Though studies have shown that the use of burs for restorative repair is comparable to that of lasers, ^24^ the smear layer produced after the use of burs could have also contributed to the poor repair bond strength.

Further investigations should focus on the analysing the failure modes between the aged and new alkasite based restorative material.

## Conclusions

Based on the results of the present study, it can be concluded that repair of alkasite based restorative material with the use of 2-step etch and rinse adhesive resulted in superior bond strength. However, surface roughening techniques such as using a bur or laser irradiation did not show significant improvement in the bond strength.

## Data Availability

Underlying data is available in Figshare at the following link: https://doi.org/10.6084/m9.figshare.25488814.v2. ^12^ Data are available under the terms of the Creative Commons Zero “No rights reserved” data waiver (CC0 1.0 Public domain dedication).
