# Supplementary material for: Effect of different surface treatments on the repair bond strength between aged and new alkasite based restorative material
Source: F1000Res. 2024 Jun 6;13:319. Originally published 2024 Apr 23. [Version 2] doi: 10.12688/f1000research.148326.2 (PMC11193086; doi:10.12688/f1000research.148326.2)
Supplement: Peer review history

# Reviewer report: Reviewer response for version 2

**DOI:** 10.5256/f1000research.167469.r287256

**Published:** 2024-06-21

**Authors:** Nazmiye Donmez

**Affiliations:** 1 Restorative Dentistry, Bolu Abant İzzet Baysal University, Bolu, Holy, Turkey

The author has corrected his article based on the reviewer's suggestions.

 I believe this manuscript paper is suitable for indexing  in F1000 Research.

Is the work clearly and accurately presented and does it cite the current literature?

Yes

If applicable, is the statistical analysis and its interpretation appropriate?

Yes

Are all the source data underlying the results available to ensure full reproducibility?

Yes

Is the study design appropriate and is the work technically sound?

Partly

Are the conclusions drawn adequately supported by the results?

Yes

Are sufficient details of methods and analysis provided to allow replication by others?

Partly

Reviewer Expertise:

dental restorative materials, fiber-reinforced composite restorations

I confirm that I have read this submission and believe that I have an appropriate level of expertise to confirm that it is of an acceptable scientific standard.

# Reviewer report: Reviewer response for version 1

**DOI:** 10.5256/f1000research.162622.r274359

**Published:** 2024-05-30

**Authors:** Nazmiye Donmez

**Affiliations:** 1 Restorative Dentistry, Bolu Abant İzzet Baysal University, Bolu, Holy, Turkey

Thank you for the opportunity to review this manuscript. This present study provides useful information about the repair protocol of alkasite restorative material. However, there are several points that need to be corrected in this manuscript. Some of the changes are defined below.

 Methods section:

- “A total of 48 acrylic blocks (4 cm height _ 2 cm width) were prepared using a self-cure acrylic resin.” Sample sizes are written incorrectly in the method section.
- “The specimens were then stored in distilled water at 37°C for one month.” Please add a reference to this sentence.
- “Group III (ASB2): The surface of alkasite based material was treated with 2 step etch and rinse adhesive where the...” Please write the brand of the adhesive.
- “Specimen preparation”: After the completion of the surface preparation of alkasite based material as described above, a freshly mixed alkasite based material as per the manufacturer’s instructions was built up to a height of 4 mm. Subsequently, the material was cured using LED light curing for 40 s.  
*The second paragraph of the Method section states that the samples were polymerized for 20 seconds. In this sentence, it is stated that it was polymerized for 40 seconds. Polymerization times were used differently in the preparation of the samples. Write the reason*.
- Add a table where the contents of the adhesives used and the alkasite material, which is the filling material, are written.
- After the bonding test, fracture-type analysis of the sample surfaces should be performed and supported by SEM.

Is the work clearly and accurately presented and does it cite the current literature?

Yes

If applicable, is the statistical analysis and its interpretation appropriate?

Yes

Are all the source data underlying the results available to ensure full reproducibility?

Yes

Is the study design appropriate and is the work technically sound?

Partly

Are the conclusions drawn adequately supported by the results?

Yes

Are sufficient details of methods and analysis provided to allow replication by others?

Partly

Reviewer Expertise:

dental restorative materials, fiber-reinforced composite restorations

I confirm that I have read this submission and believe that I have an appropriate level of expertise to confirm that it is of an acceptable scientific standard, however I have significant reservations, as outlined above.

# Response

**Published:** 2024-06-03

**Authors:** Tina Purayil

**Affiliations:** Manipal University, India

Methods ****section:

 Query 1“A total of 48 acrylic blocks (4 cm height _ 2 cm width) were prepared using a self-cure acrylic resin.” Sample sizes are written incorrectly in the method section.

 Response1:

 A total of 48 acrylic blocks (4 cm height × 2 cm width) were prepared using a self-cure acrylic resin. One-day after the polymerization of the acrylic blocks, a cylindrical hole of 4 mm × 4 mm was drilled on one side of the acrylic block. These holes were restored with alkasite based material. Hence, sample size was 48 alkasite based material blocks. These 48 blocks underwent surface preparation followed by restoration with newly added alkasite based material and were subsequently subjected to repair bond strength assessment.

 Query 2: The specimens were then stored in distilled water at 37°C for one month.” Please add a reference to this sentence.

 Response 2: The following reference was added

 Padipatvuthikul P, Mair LH. Bonding of composite to water aged composite with surface treatments. Dent Mater. 2007 Apr;23(4):519-25. 

 Query 3: Group III (ASB2): The surface of alkasite based material was treated with 2 step etch and rinse adhesive where the...” Please write the brand of the adhesive.

 Response 3: Manufacturing details have been added to the article; Adper Single Bond 2, 3M ESPE St Paul MN, USA

 Query 4: “Specimen preparation”: After the completion of the surface preparation of alkasite based material as described above, a freshly mixed alkasite based material as per the manufacturer’s instructions was built up to a height of 4 mm. Subsequently, the material was cured using LED light curing for 40 s.  

*The second paragraph of the Method section states that the samples were polymerized for 20 seconds. In this sentence, it is stated that it was polymerized for 40 seconds. Polymerization times were used differently in the preparation of the samples. Write the reason*.

 Response 4: Apologies for the error. This was due to an oversight. All samples were polymerised for 20 seconds. The manuscript has been edited to reflect this.

 Query 5: Add a table where the contents of the adhesives used and the alkasite material, which is the filling material, are written.

 Response 5: Added a table with materials used in the study along with their compositions

 Query 6: After the bonding test, fracture-type analysis of the sample surfaces should be performed and supported by SEM.

 Response 6: Thank you for your insight. We acknowledge this as a limitation of our study. As these methods were resource intensive and expensive, we could not perform the same. We will plan future experiments including SEM.

# Reviewer report: Reviewer response for version 1

**DOI:** 10.5256/f1000research.162622.r274360

**Published:** 2024-05-25

**Authors:** Emad Farhan Alkhalidi

**Affiliations:** 1 College Of Dentistry, University Of Mosul, Mosul, Iraq

Comments to author(s)

 Dear authors, 

 The subject is of interest but there are some points should be corrected.

 Title:

 Its better to be as "Effect of different surface treatments on bond strength of repairing alkasite based restorative material".

 Abstract: 

 The background needs rephrasing for "This study investigates various surface treatment methods to assess shear bond strength between set Cention N (alkasite-based restorative material) and fresh alkasite based restorative material.(mention N)."

 Method l:

 line 9; Please write the statistics test in sequential manner 

 Results:

 Rephrase, "this is conclusion"

 Methodology

 Rephrase first 3 lines, remove "aging to the acrylic block". This is not your main work. It causes confusion to reader. Put "aging" in line 8, "the specimens were aging (stored in distilled water at 37c for one month)."

 Throughout the paper write manufacturing details for materials and devices.

 Rewrite statistical analysis tests in sequential manner.

 Reference 11 in statistical analysis referred to what results?

 Line 1- "one way ANOVA showed that there was Levene’s test showed" you don't write it before

 Please check the tests of statistical analysis.

 Table 2 needs more details and explanations.

 References: 

 Ref 12, 13 not present in the article. Please check it.

Is the work clearly and accurately presented and does it cite the current literature?

Partly

If applicable, is the statistical analysis and its interpretation appropriate?

Partly

Are all the source data underlying the results available to ensure full reproducibility?

Partly

Is the study design appropriate and is the work technically sound?

Yes

Are the conclusions drawn adequately supported by the results?

Yes

Are sufficient details of methods and analysis provided to allow replication by others?

Yes

Reviewer Expertise:

conservative dentistry, endodontics, crown and bridge

I confirm that I have read this submission and believe that I have an appropriate level of expertise to confirm that it is of an acceptable scientific standard.

# Response

**Published:** 2024-06-03

**Authors:** Tina Purayil

**Affiliations:** Manipal University, India

Thanks for the comments. The following edit was done in the manuscript

 Query1: Title: Its better to be as "Effect of different surface treatments on bond strength of repairing alkasite based restorative material".

 Response 1: Title has been updated accordingly to maintain clarity and integrity “Effect of different surface treatments on the repair bond strength between aged and new alkasite based restorative material”

 Query 2 Abstract: The background needs rephrasing for "This study investigates various surface treatment methods to assess shear bond strength between set Cention N (alkasite-based restorative material) and fresh alkasite based restorative material. (mention N)."

 Response 2: Abstract background has been rephrased as suggested. To avoid confusion, we have replaced the word “fresh” with “new”

 Revised text-This study investigates various surface treatment methods to assess shear bond strength between set Cention N (alkasite-based restorative material) and new alkasite based restorative material.

 Query 3 Method: line 9; Please write the statistics test in sequential manner. 

 Response 3: Shapiro-Wilk and Levene's tests were used to check normality and Homogeneity of variance.

 Query 4 :Results: Rephrase, "this is conclusion"

 Response 4: The sentence has been edited to “Using a 2-step etch and rinse adhesive resulted in a higher repair bond strength (26.05±2.12) compared to other surface treatments. In contrast, roughening of the surface with burs led to lowest repair bond strength (17.06±3.29) (P=0.02)”

Methodology

  Query 5: Rephrase first 3 lines, remove "aging to the acrylic block". This is not your main work. It causes confusion to reader. Put "aging" in line 8, "the specimens were aging (stored in distilled water at 37c for one month)."

 Response 5: First three lines has been rephrased and aging repositioned as suggested.

 Revised text-One-day after the polymerization of the acrylic blocks, a cylindrical hole of 4 mm × 4 mm was drilled on one side of the acrylic block.

 The specimens were then stored in distilled water at 37°C for one month for aging.

 Query 6: Throughout the paper write manufacturing details for materials and devices.

 Response 6: Manufacturing details for materials and devices have been added

 Self-cure acrylic resin (DPI-RR Cold Cure, DPI, Mumbai, India)

 High speed handpiece (NSK M25, Nakanishi Inc, Kanuma City, Japan)

 Query 7: Rewrite statistical analysis tests in sequential manner.

 Response 7: We have edited it as follows:

 “Data were analyzed using SPSS version 20 (IBM Corp. Released 2011. IBM SPSS Statistics for Windows, Version 20.0. Armonk, NY: IBM Corp.). Statistical significance was set at P<0.05. Normality was tested using the Shapiro-Wilk test. Based on the normality, and test of homogeneity of the variances (Levene statistic) mean were compared using ANOVA with post-hoc Games-Howell test. Subsequently, a two-way ANOVA with post-hoc Bonferroni test for inter-group comparisons was performed to evaluate the role of surface preparation and adhesive application on the repair bond strength.”

 Query 8: Reference 11 in statistical analysis referred to what results?

 Response 8: Reference 11 is the underlying raw data of this research as per journal guidelines.

 Query 9: Line 1- "one way ANOVA showed that there was Levene’s test showed" you don't write it before. Please check the tests of statistical analysis.

 Response 9: This has been edited as per the comment in Query7.

 Query 10: Table 2 needs more details and explanations.

 Response 10: Table 2 has been revised with more details and explanation

References:

 Query 11: Ref 12, 13 not present in the article. Please check it.

 Response 11: Reference 12 and 13 were cited in the first paragraph of the discussion
